# 1742. Predictors of Antiviral Receipt for Influenza in Hospitalized U.S. Children, New Vaccine Surveillance Network (NVSN), 2016–2020

**DOI:** 10.1093/ofid/ofad500.1573

**Published:** 2023-11-27

**Authors:** Justin Z Amarin, Olla Hamdan, James W Antoon, Andrew J Spieker, Laura S Stewart, Marian G Michaels, John V Williams, Eileen J Klein, Janet A Englund, Geoffrey A Weinberg, Peter G Szilagyi, Jennifer E Schuster, Rangaraj Selvarangan, Julie A Boom, Flor M Munoz, Mary A Staat, Elizabeth P Schlaudecker, James Chappell, Benjamin R Clopper, Heidi L Moline, Angela P Campbell, Samantha M Olson, Natasha B Halasa

**Affiliations:** Vanderbilt University Medical Center, Nashville, Tennessee; Vanderbilt University Medical Center, Nashville, Tennessee; Vanderbilt University Medical Center, Nashville, Tennessee; Vanderbilt University Medical Center, Nashville, Tennessee; Vanderbilt University Medical Center, Nashville, Tennessee; UPMC Children's Hospital of Pittsburgh, Pittsburgh, Pennsylvania; University of Pittsburgh, Pittsburgh, Pennsylvania; University of Washington School of Medicine, Seattle, Washington; Seattle Children’s Hospital, Seattle, Washington; University of Rochester School of Medicine & Dentistry, Rochester, NY; UCLA School of Medicine, Agoura Hills, California; Children’s Mercy Kansas City, Kansas City, Missouri; Children’s Mercy Kansas City, Kansas City, Missouri; Texas Children’s Hospital, Houston, Texas; Baylor College of Medicine, Houston, TX; Cincinnati Children’s Hospital Medical Center, Cincinnati, Ohio; Cincinnati Children's Hospital Medical Center, Cincinnati, Ohio; Vanderbilt University Medical Center, Nashville, Tennessee; US Centers for Disease Control & Prevention, Buffalo, New York; Centers for Disease Control and Prevention, Atlanta, Georgia; Centers for Disease Control and Prevention, Atlanta, Georgia; Centers for Disease Control and Prevention, Atlanta, Georgia; Vanderbilt University Medical Center, Nashville, Tennessee

## Abstract

**Background:**

Children hospitalized with suspected or confirmed influenza should promptly receive influenza antivirals, as recommended by the Infectious Diseases Society of America, the American Academy of Pediatrics, and the Centers for Disease Control and Prevention (CDC). However, despite these recommendations, antiviral receipt remains suboptimal. We assessed predictors of antiviral receipt for influenza in hospitalized children.

**Methods:**

We conducted active, population-based surveillance of children presenting with fever or respiratory symptoms from December 1, 2016, to March 31, 2020, at seven children’s hospitals in the CDC NVSN. The cohort consisted of children hospitalized with influenza confirmed by research molecular testing. We assessed predictors of antiviral receipt, selected *a priori*, using logistic regression, and included all in the final model.

**Results:**

We enrolled and tested 16,853 hospitalized children for influenza, identifying 1,120 laboratory-confirmed cases (6.6%). Of these laboratory-confirmed cases, clinicians ordered influenza testing for 693/1,120 (61.9%), and among those tested, 599/693 (86.4%) tested positive by clinical assays. Overall, 545/1,120 children (48.7%) received influenza antivirals. Of those with a positive clinical test, 405/599 (67.6%) received antivirals. Factors associated with higher odds of antiviral receipt included an underlying oncologic or immunocompromising disorder, symptom onset ≤ 2 days, intensive care unit (ICU) admission at presentation, use of influenza antivirals for this illness prior to hospitalization, and a positive clinical test (**Table**). Additionally, we found that a negative clinical test was associated with lower odds of antiviral receipt, and receipt varied significantly by study site.

Table

**Figure:**
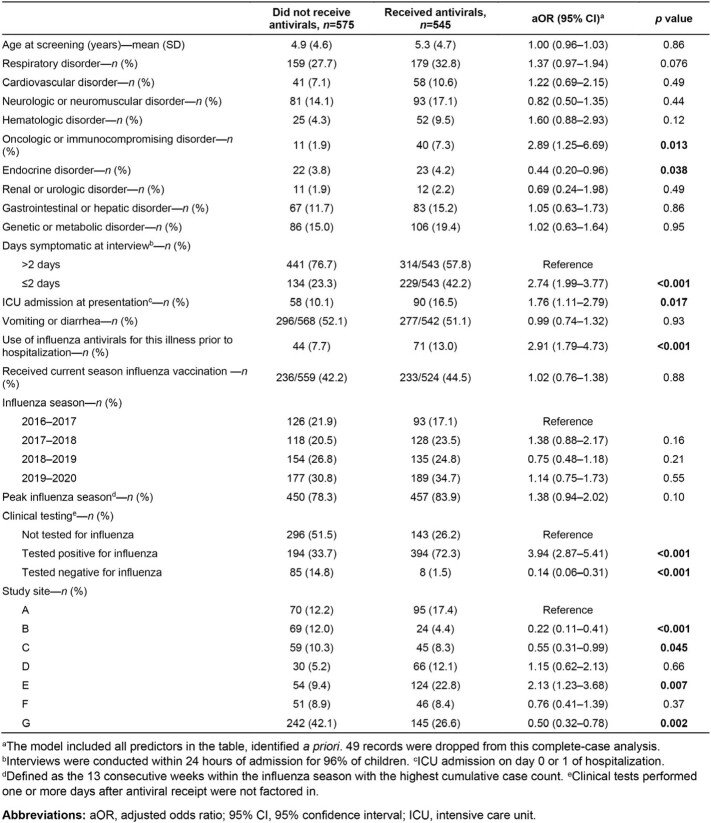

Logistic regression model of influenza-specific antiviral receipt among hospitalized children testing positive for influenza by research testing, December 1, 2016, and March 31, 2020 (N=1,120).

**Conclusion:**

More than half of children hospitalized with influenza in our surveillance population did not receive antivirals, despite treatment recommendations. Efforts to improve antiviral receipt are important for optimizing care in all children hospitalized with influenza, and clinician education should continue to highlight the use of antivirals across the full spectrum of children hospitalized with influenza, including children not admitted to the ICU and those without underlying conditions.

**Disclosures:**

**Marian G. Michaels, MD, MPH**, Merck: Grant/Research Support|Viracor: Grant/Research Support **John V. Williams, MD**, Merck: Grant/Research Support|Quidel: Board Member **Janet A. Englund, MD**, Ark Biopharma: Advisor/Consultant|AstraZeneca: Advisor/Consultant|AstraZeneca: Grant/Research Support|GlaxoSmithKline: Grant/Research Support|Meissa Vaccines: Advisor/Consultant|Merck: Grant/Research Support|Moderna: Advisor/Consultant|Moderna: Grant/Research Support|Pfizer: Advisor/Consultant|Pfizer: Grant/Research Support|Sanofi Pasteur: Advisor/Consultant **Geoffrey A. Weinberg, MD**, Merck & Co: Honoraria **Rangaraj Selvarangan, BVSc, PhD, D(ABMM), FIDSA, FAAM**, Abbott: Honoraria|Altona Diagnostics: Grant/Research Support|Baebies Inc: Advisor/Consultant|BioMerieux: Advisor/Consultant|BioMerieux: Grant/Research Support|Bio-Rad: Grant/Research Support|Cepheid: Grant/Research Support|GSK: Advisor/Consultant|Hologic: Grant/Research Support|Lab Simply: Advisor/Consultant|Luminex: Grant/Research Support **Flor M. Munoz, MD, MSc**, CDC respiratory virus surveillance: Grant/Research Support|Gilead: Grant/Research Support|Moderna, sanofi, aztra zeneca, Merck, GSK: Advisor/Consultant|NIH: DSMB|NIH COVID-19 vaccines in pregnancy: Grant/Research Support|Pfizer Pediatric COVID-19 vaccines: Grant/Research Support|Pfizer, Dynavax, Monderna, Meissa, NIH: DSMB **Mary A. Staat, MD, MPH**, CDC: Grant/Research Support|Cepheid: Grant/Research Support|Merck: Grant/Research Support|NIH: Grant/Research Support|Pfizer: Grant/Research Support|Up-To-Date: Honoraria **Elizabeth P. Schlaudecker, MD, MPH**, Pfizer: Grant/Research Support|Sanofi Pasteur: Advisor/Consultant **Natasha B. Halasa, MD, MPH**, Merck: Grant/Research Support|Quidell: Grant/Research Support|Quidell: donation of kits|Sanofi: Grant/Research Support|Sanofi: vaccine support

